# Decision-making and referral processes for patients with motor neurone disease: a qualitative study of GP experiences and evaluation of a new decision-support tool

**DOI:** 10.1186/s12913-017-2286-0

**Published:** 2017-05-08

**Authors:** Susan Baxter, Christopher J. McDermott

**Affiliations:** 1 School of Health and Related Research, University of Sheffield, Regent Court, 30 Regent Street, Sheffield, UK; 2 Sheffield Institute for Translational Neuroscience, University of Sheffield, Sheffield, UK

**Keywords:** Motor neuron disease, Amyotrophic lateral sclerosis, Consultation and referral, Differential diagnosis

## Abstract

**Background:**

The diagnosis of motor neurone disease (MND) is known to be challenging and there may be delay in patients receiving a correct diagnosis. This study investigated the referral process for patients who had been diagnosed with MND, and whether a newly-developed tool (The Red Flags checklist) might help General Practitioners (GPs) in making referral decisions.

**Methods:**

We carried out interviews with GPs who had recently referred a patient diagnosed with MND, and interviews/surveys with GPs who had not recently referred a patient with suspected MND. We collected data before the Red Flags checklist was introduced; and again one year later. We analysed the data to identify key recurring themes.

**Results:**

Forty two GPs took part in the study. The presence of fasciculation was the clinical feature that most commonly led to consideration of a potential MND diagnosis. GPs perceived that their role was to make onward referrals rather than attempting to make a diagnosis, and delays in correct diagnosis tended to occur at the specialist level. A quarter of participants had some awareness of the newly-developed tool; most considered it useful, if incorporated into existing systems.

**Conclusions:**

While fasciculation is the most common symptom associated with MND, other bulbar, limb or respiratory features, together with progression should be considered. There is a need for further research into how decision-support tools should be designed and provided, in order to best assist GPs with referral decisions. There is also a need for further work at the level of secondary care, in order that referrals made are re-directed appropriately.

**Electronic supplementary material:**

The online version of this article (doi:10.1186/s12913-017-2286-0) contains supplementary material, which is available to authorized users.

## Background

Motor neurone disease (MND), which may also be termed Amyotrophic Lateral Sclerosis (ALS), is one of the most common neurodegenerative conditions of adult life. There is an estimated annual incidence of 2 in 100,000, and prevalence of 5–7 per 100,000 in most countries [1]. The disease causes progressive paralysis, with typically a three to five year survival period following diagnosis [2]. MND has varying forms of presentation, with three recognised patterns of limb, bulbar or respiratory onset [3].

The diagnosis of MND has been described as being often fraught with difficulties [4]. Clinical signs may mimic several other neurological syndromes [5]. There may be a failure to recognize the significance of symptoms amongst patients, carers and primary and secondary care health professionals [4]. For general practitioners (GPs), this is in part due to the fact that most will only see one or two cases in their career. One UK study reported a mean time from onset to diagnosis of 16.2 months, with delays due to incorrect diagnosis, not considering the diagnosis, not identifying the symptoms as having a neurological cause, or referring to non-neurology specialist services [6].

The Motor Neurone Disease Association, in conjunction with The Royal College of General Practitioners and specialist neurologists developed a Red Flag tool for MND in 2014 (see Additional file 1). The tool was developed via a series of meetings of MND specialists. It comprises a checklist of symptoms that may indicate MND, and was designed to respond to concerns regarding delays in patients receiving specialist input. It was hoped that the tool may improve timely referrals to neurology, and speed up the time to accurate diagnosis [7].

## Methods

The aim of the current study was to investigate the referral and subsequent journey of patients who are later diagnosed with MND. We explored GP decision-making processes, and whether the newly-developed checklist for GPs might assist in optimal referral of patients with muscle weakness.

We used qualitative interview methods and collected data at two time points: prior to issuing of the Red Flags toolkit; and a year following its introduction. We recruited GPs who had referred a patient within the last 12 months who had been subsequently diagnosed with MND, and those who had not. We used two methods of recruitment. Firstly, contact details for GPs that had recently referred, were provided by the regional MND centres, and they were invited to take part in telephone interviews via letter. Secondly, recruitment was also sought via an in-person approach at national and regional GP events. During the second phase of the study, we invited GPs at a training event (not related to neurological disorders) to complete a brief questionnaire to supplement the interview data.

### Ethics, consent and permissions

The study was performed in accordance with the Declaration of Helsinki. It received ethical approval from the School of Health and Related Research Ethical Committee at the University of Sheffield (reference 000884). An information sheet was provided to participants prior to their agreeing to be interviewed as part in the study. A consent form was returned by post/email prior to the interviews. The questionnaire contained brief information about the study, with completion and return taken as consent to participate.

#### Data collection and analysis

Semi-structured interviews were carried out over the telephone by the first author, an experienced qualitative researcher who had carried out previous studies in the field of MND. Data were collected during the periods of October 2013 to January 2014, and January to April 2015, after publicising of the Motor Neurone Disease Association (MNDA) Red Flag tool in early 2014. Participants had no prior knowledge of the researcher. The semi-structured content of the interviews was based on an interview schedule which was piloted in one interview before use. The 20–30 min interviews were recorded, transcribed and analysed using techniques of thematic analysis whereby interview transcripts are read line by line and concepts (or themes) identified and assigned a code [8]. Recorded data were deleted immediately following transcription, and transcriptions were anonymised prior to analysis. Data within each code were examined and compared to develop a “tree” of themes and sub–themes across the interviews. For example extracts describing GPs use of websites were grouped and labelled as a subtheme “sources of knowledge”, within the overarching theme of “factors influencing differential diagnosis”. Analysis was undertaken by the first author, emerging themes were discussed with the second author, and drafts were circulated to the Red Flags group for comment. Atlas.ti software (version 7, Scientific Software Development) was used to support the systematic process of data coding and retrieval. The questionnaire used in phase two sought information on awareness of the tool (Additional file 2). Reporting of the study adheres to COREQ guidelines.

## Results

**Fig. 1:**
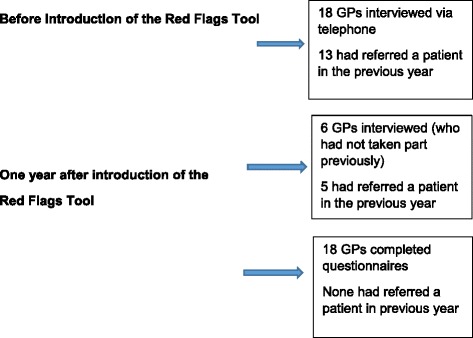
The recruitment process

Forty two GPs took part in the study. Figure 1 provides an overview of the recruitment process. Eighteen were interviewed before introduction of the tool and six were interviewed at the second time point. We achieved a 19% response rate for participants who had recently referred (18 of 108 approached by letter). We are unable to calculate a precise response rate for those who had not referred, as we used our networks of contacts to recruit, publicised the study at a national conference with many hundreds of delegates, and many other events and training where GPs were present, with limited success. We estimate that the six participants were recruited from several hundred approaches. Eighteen GPs at a training event in Yorkshire (where there were 200–250 attendees) completed questionnaires at the second point of data collection.

The sample consisted of 19 males and 23 females; four with fewer than five years’ experience as a GP, fourteen with five to ten years’ experience, and 23 with more than ten years’ experience. Three GPs reported that they had not seen any patients with MND during their career, 23 estimated that they had seen one to three patients, and 16 estimated that they had seen more than three.

Analysis of the interview transcripts identified four main themes in the data: uncertainty; symptoms of concern; factors influencing differential diagnosis; and referral processes and systems (see Table 1). In the following paragraphs these themes and subthemes will be described, with a number of illustrative quotes provided.

**Table 1:** Themes and subthemes in the data

Theme	Subthemes
1. Uncertainty	
2. Symptoms of concern	Fasciculation
	Progression
	Speech disturbance
	Lack of sensory involvement
	Context
3. Factors influencing differential diagnosis	Familiarity with patient
	Influence of patient delay
	Sources of GP knowledge
4. Referral processes and systems	Discussion with patient
	Referral pathways
	Personal contact

### Uncertainty

The most common description of patients who had been referred, or those that were likely to be referred by GPs was of a patient who “just did not fit” any familiar presentation:

I couldn’t make him fit any of my things I knew something about. (GP4)

GPs described their feeling of uncertainty, a pattern that they were unable to explain:

It didn’t add up and it wasn’t like ME. It was sort of more intuitive I thought there’s something not right here, very not right here. (GP2)

As a result of this uncertainty participants described a tendency to categorise patients as a general “neurological problem”. There was a recognition that limited knowledge of the disease meant that GPs relied on specialist opinion. The priority was perceived to be referring the patient on to the expert, rather than attempting to make a differential diagnosis:

I felt I had to make the referral whatever, you know I didn’t feel that the referral was going to be dependent on seeking more information, I felt this is somebody who needs to be seen. (GP6)

Participants reflected on whether MND had been at the forefront of their mind when they had referred the patient, in most cases it had not.

I was worried it was neurodegenerative. That much I was aware of so that is something. I would love to say the top of my list was MND, but I have to be honest that it wasn’t. (GP4)

### Symptoms of concern

Participants were asked to describe the main symptoms that had either caused them concern, or would make them concerned if seen in a patient. Participants identified *fasciculation* as the most common sign:

It was the fasciculation more than the weakness obviously that was the real red flag to my mind. (GP5)

*Disease progression* was identified as a key symptom of concern by eight participants:

Her story was not compatible with a stroke, it was obviously one of sort of progressive weakness rather than sudden weakness. (GP3)

Three GPs mentioned the presence of *speech problems* as a trigger for neurology referral. This knowledge was based on personal experience for two:

Speech in most cases that I’ve seen has been involved very early on. (GP8)

Oh yes the other thing about this lady is that her voice was a bit affected as well. No it wasn’t croaky it was just that she hadn’t got such a strong voice. (GP2)

Other participants did not include speech in their consideration of possible symptoms, and one GP perceived speech disturbance as a later onset feature:

Well I’d hope that it’d be diagnosed before they got swallowing or speech problems. (GP15)

Two GPs highlighted that a lack of *sensory involvement* would be important in the clinical pattern:

Yes I think it was just kind of very clear weakness was the one thing without any sort of sensory problems as well. (GP1)

One participant described the *context* of the presenting symptoms as important in understanding the overall picture:

It’d be the context of the muscle weakness so, it would depend on the patient’s history, you know their story of what had happened. (GP11)

### Factors influencing differential diagnosis

*Familiarity with the patient* was highlighted as a potentially important element in a GP’s ability to assess the pattern of presentation and disease progression:

If you knew somebody you know what is different. I don’t know if one of my colleagues had seen him from that if they would have been as aware. (GP1)

GPs described four cases where there had been *patient delay* seeking medical input, and this had made symptoms more noticeable. Patients had either described experiencing symptoms for several months but did not make an appointment, or had delayed making follow up appointments following a consultation:

I think it would have been a very different situation if it had been a patient presenting with early symptoms that were quite vague. (GP10)

During the interviews we explored the *sources of GP knowledge* about MND. While some participants described having several patients with MND at their practice, for others their knowledge of MND was based on their initial training:

I think Motor Neurone Disease had crossed my mind because I had seen fasciculation and I think obviously going back to medical school, that is the one thing that you kind of tie with it. (GP14)

Participants were asked what resources they would use if they had a patient with symptoms of muscle weakness. Some GPs were unable to identify any resources, others described using text books or speaking to colleagues. The most commonly mentioned resources were online: GP Notebook; patient.co.uk; or a general search using Google:

I suppose, well I guess I would start looking online. Looking up you know GP Notebook or something like that…you know causes of a bulbar palsy. (GP4)

Participants were asked to describe any new resources that would be helpful. While the majority were not able to suggest any, one GP suggested the use of video clips similar to YouTube, another a brief leaflet, and several suggested embedding additional information in the resources they were already using such as GP Notebook:

Just little 3–5 min video of one of the consultants or registrars, or you know someone relatively charismatic to do a little video with a patient and to video those fasciculation’s….put it on something like a link, a lot of us use something called GP notebook…you know a link from there or other GPs use Mentor or links from our computer systems. (GP15)

### Referral processes and systems

GPs were asked about the content of their *discussion with the patient* at the time of referral. There was some variation in opinion regarding whether or not it was preferable to mention the disease to patients prior to referral. Some patients had already researched MND on the internet so GPs had been required to discuss the disease. Participants described the challenge of having to make a decision with the patient in front of them, their own lack of certainty, and also the potential impact of mentioning MND to a patient:

I know most patients think Dignitas, euthanasia and everything to do with motor neurone disease so I didn’t want to send him into orbit. (GP2)

I said ‘I’m really worried about this because you know, you’re getting worse and you shouldn’t be getting worse if it’s a stroke and I think it might be something else and I want you to be seen urgently’, I didn’t tell him what I thought it might be. (GP9)

Two participants were concerned that referral to a specialist MND clinic without a diagnosis, might be traumatic for patients:

I think if I was going to refer them to the motor neurone disease clinic I would tell them otherwise they’d be sat in the waiting room thinking ‘what the hell am I doing here?’ (GP15)

There are no examples in the data of GPs adopting or advocating a “watch and wait” approach. Patients were referred to specialists on first presentation of symptoms perceived as worrying.

Of the 18 patients who had been recently referred by participants, twelve had reportedly experienced convoluted *referral pathways* to specialist MND services. In these cases MND had not been suspected by GPs, and patients had been referred on to non-MND specialists. It was typically at this point that incorrect diagnoses had been made and inappropriate treatment commenced, or patients had been referred from specialist to specialist with no diagnosis.

Two patients had been referred to neurology and diagnosed as having had a transient ischaemic attack; and one had been referred to neurology and then on to orthopaedics. Two patients had been referred to an Ear Nose and Throat (ENT) specialist for suspected laryngeal problems, one of these was then referred on to Speech and Language Therapy. One patient had been referred to a falls clinic and then on to physiotherapy; and another directly for physiotherapy. One patient was referred to a muscular-skeletal service, another to a neurophysiologist; one to orthopaedics, one was treated by a private practitioner as having high cholesterol; and the final patient had been referred initially to a geriatrician:

So he then had an MRI of his head which showed a small lesion on the left side which corresponded with possibly some right sided symptoms, so he was told he had a stroke. (GP12)

When I examined him I was concerned initially to exclude something nasty on his larynx so I actually sent him in a 2 week wait into ENT. (GP9)

He was put on a bit of a cocktail of cholesterol drugs by somebody and was getting a lot of muscle problems which I think clouded the symptoms he had. (GP7)

For those patients referred to ENT, it was only when speech became dysarthric, rather than dysphonic that the diagnosis became questioned.

Two GPs mentioned the tendency for a diagnostic line to be pursued once it had been started:

I think sort of reflecting back on it, it’s always just a reminder to maybe ignore what’s gone before and start with a clean piece of paper. (GP7)

There was some variation in the local *referral systems* described by participants. Some reported that the system required a general neurology referral which was then triaged. There was frustration at the waiting time for neurology appointments, and that it was not possible to direct referrals to specific specialties:

You can’t do that through choose and book these days, you have to refer generally. (GP4)

You put down urgent, they decide it’s not urgent or vice versa. Once the appointment goes in, no matter what’s on it, it’s reviewed, the hospital clinic, they decide what happens. (GP8)

The benefit of *personal contact* via telephone with a specialist was highlighted by several participants:

Unfortunately I’m sure like with a lot of areas of the country we have great problems in getting urgent neurology appointments. I actually phoned up the local neurologist that I thought she was going to get seen by. (GP6)

### Awareness and views of the new Red flag MND tool

Six of the 24 participants at the second point of data collection (12–15 months after its publication) had some awareness of the new tool. One of these had been sent it as part of a pack from the MNDA following a patient diagnosis, one person had seen it in a magazine article, two people had learned of it via word of mouth, and two were unable to recall where they had heard of it. Of the 18 GPs who completed the questionnaire (which included a copy of the checklist), eleven described it as “useful”, “clear”, “easy to use” or “helpful”. One described it as useful although “they had lots of other similar checklists”, another was concerned it would result in over-referral of patients with muscle twitching, and one perceived that it “had too many words, needs to be electronic”. Four did not comment on the format.

Interview participants highlighted that a limitation of tools such as this, is that the diagnosis needs to be already in the clinician’s mind to prompt its use. Given its rarity, diagnoses other than MND may be considered more readily.

## Discussion

GPs described a range of symptoms which would prompt them to consider MND as a diagnosis, with fasciculation being the most common, and speech-related symptoms least common. Other studies have reported that patients with bulbar onset have a shorter mean time from symptoms to diagnosis [5, 6]. It was interesting therefore that speech-related symptoms were least frequently mentioned as being associated with MND. The GP role was perceived to be onward referral following the recognition that something was not right, rather than attempting to make a diagnosis. However, if the onward referral was made to a non-MND service then delays were incurred, as non-MND secondary care services often did not identify MND. Many of our participants described frustration with referral pathways which inhibited selection of specialist services, with long waiting times for neurology appointments.

Participants described relying on knowledge from medical school, speaking to colleagues, or using websites to access information. The use of the Red Flags checklist to aid referral decision-making diagnosis was welcomed, although only a quarter of our sample were aware of it. The incorporation of checklists into existing systems seems to be crucial to increase awareness and usage.

This study echoes earlier work in describing the often challenging pathway for patients before a diagnosis of MND is made [5, 6]. The findings highlight that it is following referral to non-MND specialist services that misdiagnosis/delays in diagnosis may often occur. A recent paper which reconstructed the timelines of a patient journey to a specialist multi-disciplinary clinic [9] reported that the majority of patients were seen by a general practitioner, and subsequently by neurology services. There was an average of four contacts with health services and 4.8 investigations/tests, prior to their first Clinic visit.

A review of GP diagnosis and referral by the Kings Fund [10] reported that the very low prevalence of certain conditions, and high degree of overlap in symptoms for serious and common conditions, makes diagnosis in primary care difficult. A tool that is easily accessible to GPs to help direct referrals most appropriately, therefore has potential value.

It has been estimated that an average GP would encounter a new case of MND once in their professional lifetime [11]. This rarity of GPs encountering MND was supported in our study, with just over half of the participants (23 of 42) estimating that they had seen between one and three patients with MND during their career. The Kings Fund review [9] suggested that the provision of decision-support tools may be beneficial to GPs. However, any paper-based checklist may be one of many, filed away, or not easily retrieved, particularly when a condition is rare.

A recent review of methods to manage referral between GPs and hospital specialists across all patient groups, identified inconsistent evidence for the use of guidelines or decision-support tools having an impact on referral practice [12]. The included studies evaluated use of guidelines for a range of conditions including dementia and cancer. The review highlighted multiple elements that will impact on the effectiveness of interventions aiming to change GP referrals, including factors relating to the doctor, the patient, and local processes and systems. Delay in referral has been reported for other conditions [13–15]. A study evaluating a GP decision-support tool for cancer diagnosis echoed our work in concluding that it could be of value, although usability needed further consideration [16]. Hill et al. highlighted that while referral guidelines in all areas of medicine have proliferated, their effect has sometimes been little evaluated [17].

### Strengths and limitations

Recruitment of GPs proved challenging, with a low response rate to initial approach and invitations, and large drop out following initial agreement to participate. Our final sample size, although small, is typical of qualitative studies, and we were successful in achieving diversity in terms of GP characteristics. We aimed to recruit more equal numbers of those who had referred patients versus those who had not referred. The relative rarity of the condition reportedly influenced a reluctance to devote valuable time to taking part in those who had not referred. The survey increased the number of responses from GPs who had not referred, although we acknowledge that this was a small sample size from a single region. Our sample may also contain a larger proportion of GPs who had patients with convoluted referral routes than is typical. GPs were keen to “tell the stories” of patients who had experienced delay and distress, and may have been more willing to volunteer to participate.

## Conclusions

Fasciculation is the most common symptom GPs associate with MND, and patients presenting with other bulbar, limb or respiratory features may be referred to non-MND specialist services, delaying correct diagnosis. A newly-developed checklist which includes a comprehensive list of potential signs has potential to be a useful tool for GPs, although levels of awareness and usage need to be enhanced by incorporating it into existing systems. There is a need for further research into how decision-support tools should be designed and provided, in order to best assist GPs with referral decisions. There is also a need for further work at the level of secondary care, in order that referrals made are re-directed appropriately.

## Additional files

Additional file 1: The Red Flags Checklist. Motor Neurone Disease Association. Permission to use granted by J. Bedford. Checklist for use by GPs (PDF 109 kb)

Additional file 2: Survey of views and awareness regarding the Red Flags Checklist. Questionnaire used in the study. (DOCX 115 kb)

## Abbreviations

ALS: Amyotrophic lateral sclerosis
ENT: Ear nose and throat
GP/GPs: General practitioner/general practitioners
MND: Motor neurone disease
MNDA: Motor Neurone Disease Assocation
