# Supplementary material for: Decision-making and referral processes for patients with motor neurone disease: a qualitative study of GP experiences and evaluation of a new decision-support tool
Source: BMC Health Serv Res. 2017 May 8;17:339. doi: 10.1186/s12913-017-2286-0 (PMC5422976; doi:10.1186/s12913-017-2286-0)
Supplement: Supplementary file 1 — The Red Flags Checklist. Motor Neurone Disease Association. Permission to use granted by J. Bedford. Checklist for use by GPs (PDF 109 kb) [file 12913_2017_2286_MOESM1_ESM.pdf]

motor neurone disease association

Royal College of General Practitioners

Painless, progressive weakness – Could this be Motor Neurone Disease?

1. Does the patient have one or more of these symptoms?

Bulbar features

- Dysarthria
  - Slurred or quiet speech often when tired
- Dysphagia
  - Liquids and/or solids
  - Excessive saliva
  - Choking sensation especially when lying flat
- Tongue fasciculations

Limb features

- Focal weakness
- Falls/trips – from foot drop
- Loss of dexterity
- Muscle wasting
- Muscle twitching/ fasciculations
- Cramps
- No sensory features

Respiratory features

- Hard to explain respiratory symptoms
- Shortness of breath on exertion
- Excessive daytime sleepiness
- Fatigue
- Early morning headache
- Orthopnoea

Cognitive features (rare)

- Behavioural change
- Emotional lability (not related to dementia)
- Fronto-temporal dementia

2. Is there progression?

Supporting factors

- Asymmetrical features
- Age – MND can present at any age
- Positive family history of MND or other neurodegenerative disease

Factors NOT supportive of MND diagnosis

- Bladder / bowel involvement
- Prominent sensory symptoms
- Double vision / Ptosis
- Improving symptoms

If yes to 1 and 2 query MND and refer to Neurology

If you think it might be MND please state explicitly in the referral letter.
Common causes of delay are initial referral to ENT or Orthopaedic services.

Additional resources:
MND Association downloads and publications at www.mndassociation.org/gp

Bulbar features

25% of patients present with bulbar symptoms

- Dysarthria
  - Quiet, hoarse or altered speech
  - Slurring of speech often when tired
- Dysphagia – more often liquids first and later solids. Initially can be sensation of catching in throat or choking when drinking quickly.
- Excessive saliva
- Choking sensation when lying flat
- Weak cough – often not noticed by the patient

Painless progressive dysarthria – consider neurological referral rather than ENT.

Limb features

70% of patients present with limb symptoms

- Focal weakness – painless with preserved sensation
- Distal weakness
  - Falls/trips – from foot drop
  - Loss of dexterity eg problems with zips or buttons
- Muscle wasting – hands and shoulders. Typically asymmetrical
- Muscle twitching/fasciculations
- Cramps

Respiratory features

Respiratory problems are often a late feature of MND and an unusual presenting feature. Patients present with features of neuromuscular respiratory failure

- Shortness of breath on exertion
- Excessive daytime sleepiness
- Fatigue
- Early morning headache. Patients often describe a 'muzziness' in the morning, being slow to get going or as if hung over
- Un-refreshing sleep
- Orthopnoea
- Frequent unexplained chest infections
- Weak cough and sniff
- Nocturnal restlessness and/or sweating

Consider MND if investigations for breathlessness do not support a pulmonary or cardiac cause.

Cognitive features

Frank dementia at presentation is rare. Cognitive dysfunction is increasingly recognised, as evidenced by:

- Behavioural change such as apathy or lack of motivation
- Difficulty with complex tasks
- Lack of concentration
- Emotional lability (not related to dementia)

Ask specifically about a family history of these features.

Development group for this resource:
RCGP (L Davies, R Pizzaro-Duhart, I Rafi) **MND Association** (J Bedford, H Fairfield)
Neurology (P Callagher, C McDermott, K Morrison, R Orrell, A Radunovic, S Weatherby, A Wills) **Palliative Medicine** (I Baker)

© Copyright 2014
