# Supplementary material for: Decision-making and referral processes for patients with motor neurone disease: a qualitative study of GP experiences and evaluation of a new decision-support tool
Source: BMC Health Serv Res. 2017 May 8;17:339. doi: 10.1186/s12913-017-2286-0 (PMC5422976; doi:10.1186/s12913-017-2286-0)
Supplement: Supplementary file 2 — Survey of views and awareness regarding the Red Flags Checklist. Questionnaire used in the study. (DOCX 115 kb) [file 12913_2017_2286_MOESM2_ESM.docx]

**Appendix 2. Interview topic guide and questionnaire**

**Interview topic guide**

**First time point**

1. Check understanding of participant information sheet, willingness to participate, aim of discussion, and assurances regarding confidentiality.

2. Participant characteristics: gender; age band (under 40, over 40), years practice as a GP (less than 5, 5-10, more than 10); number of patients with MND during career (none, 1-2, 3 or more).

*GPs who have referred*

3. For the patient that you recently referred to a neurologist with muscle weakness can you tell me about the pathway of care:

- What signs/symptoms did the patient present with?

- Which of these were of more/less concern to you?

- Can you remember what would have been discussed with the patient at the first visit?

- What were the thoughts and decision-process that you went through at this appointment? What was the reason you referred when you did? Did you have any uncertainties, if so what were they?

- Did you order any tests?

- Did you seek information or advice from anywhere? What did you think of any information you obtained/used? What information/advice would be helpful?

- What subsequently happened?

- What was the interval between first presentation and referral?

*GPs who have not referred*

3. If you have a patient presenting with muscle weakness what would be the process that you would follow?

- What signs/symptoms might a patient present with?

- Which symptoms would be of more/less concern to you?

- What would you discuss with the patient at a first consultation?

- What would your thinking and decision-making process be? What would make you think of motor neurone disease? At what point would you refer?

- Would you order any tests?

- Would you seek information or advice from anywhere? What information/advice would be helpful?

- What would be a typical pathway for a patient to follow?

**Second timepoint**

*GPs who have referred*

1. Check understanding of participant information sheet, willingness to participate, aim of discussion, and assurances regarding confidentiality.

2. Have you referred any patients with muscle weakness in the last 12 months?

3. If you have, what was the pathway that the patient followed?

- What signs/symptoms did the patient present with?

- Which of these were of more/less concern to you?

- Can you remember what would have been discussed with the patient at the first visit?

- What were the thoughts and decision-process that you went through at this appointment? What was the reason you referred when you did? Did you have any uncertainties, if so what were they?

- Did you order any tests?

- Did you seek information or advice from anywhere? What did you think of it? What information/advice would be helpful?

- What subsequently happened?

- What was the interval between first presentation and referral?

- If not mentioned – have you heard of the Red Flags checklist – have you seen it – have you used it – if you have what do you think of it?

*GPs who have not referred*

- What signs/symptoms might a patient present with?

- Which symptoms would be of more/less concern to you?

- What would you discuss with the patient at a first consultation?

- What would your thinking and decision-making process be? What would make you think of motor neurone disease? At what point would you refer?

- Would you order any tests?

- Would you seek information or advice from anywhere? What information/advice would be helpful?

- What would be a typical pathway for a patient to follow?

- If not mentioned – have you heard of the Red Flags checklist – have you seen it – have you used it – if you have what do you think of it?

**Questionnaire**


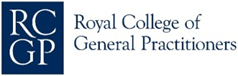

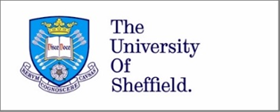

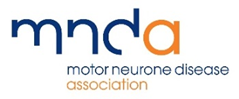


**Would you be willing to take part in a research study about referral pathways for patients with muscle weakness?**

The Royal College of General Practitioners introduced the Red Flags Toolkit in December 2013, to prompt GPs to consider motor neurone disease as a possible diagnosis when patients present with muscle weakness.

The Motor Neurone Disease Association are funding us to carry out an evaluation of this resource.

We are researching the level of awareness and views of this toolkit amongst GPs.

Responses are entirely anonymous.

The survey will take a few minutes to complete.

Dr Susan Baxter

School of Health and Related Research

University of Sheffield

Regent Court

30 Regent Street

Sheffield S14DA

V1 23/4/15

**RED FLAGS FOR MUSCLE WEAKNESS RESEARCH STUDY**

1. Are you? Male Female

2. For how many years have you practiced as a GP?

Less than 5 years Five to ten years More than ten years

3. How many patients with motor neurone disease have you seen over the course of your career?

None One to three More than three

4. Were you aware of the Royal College of General Practitioners Red Flags Toolkit/checklist before today?

Yes No

5. If you were aware of it, can you remember how you first came across it?

6. The Red Flags for Muscle Weakness checklist is provided overleaf.

What is your opinion of it? Is it useful? Any comments on the format?

MANY THANKS FOR TAKING PART AND GIVING US YOUR VIEWS
